# Estradiol-inducible AvrRps4 expression reveals distinct properties of TIR-NLR-mediated effector-triggered immunity

**DOI:** 10.1093/jxb/erz571

**Published:** 2020-02-12

**Authors:** Bruno Pok Man Ngou, Hee-Kyung Ahn, Pingtao Ding, Amey Redkar, Hannah Brown, Yan Ma, Mark Youles, Laurence Tomlinson, Jonathan D G Jones

**Affiliations:** 1 The Sainsbury Laboratory, University of East Anglia, Norwich Research Park, Norwich, UK; 2 Department of Genetics, University of Córdoba, Córdoba, Spain; 3 University of Essex, UK

**Keywords:** Cell death, defence gene expression, estradiol-inducible expression system, Golden Gate modular cloning, hypersensitive response, MAP kinase, NLR activation, plant innate immunity, protein complex

## Abstract

Plant nucleotide-binding domain, leucine-rich repeat receptor (NLR) proteins play important roles in recognition of pathogen-derived effectors. However, the mechanism by which plant NLRs activate immunity is still largely unknown. The paired Arabidopsis NLRs RRS1-R and RPS4, that confer recognition of bacterial effectors AvrRps4 and PopP2, are well studied, but how the RRS1/RPS4 complex activates early immediate downstream responses upon effector detection is still poorly understood. To study RRS1/RPS4 responses without the influence of cell surface receptor immune pathways, we generated an Arabidopsis line with inducible expression of the effector AvrRps4. Induction does not lead to hypersensitive cell death response (HR) but can induce electrolyte leakage, which often correlates with plant cell death. Activation of RRS1 and RPS4 without pathogens cannot activate mitogen-associated protein kinase cascades, but still activates up-regulation of defence genes, and therefore resistance against bacteria.

## Teaser

Inducible expression of AvrRps4 activates RRS1/RPS4-mediated effector-triggered immunity without the presence of pathogens, allowing us to characterize downstream immune responses triggered by TIR-NLRs without cell surface receptor-mediated immunity.

## Introduction

To investigate plant immunity, researchers routinely conduct pathogen inoculations on plants in a controlled environment. Upon pathogen attack, plants activate innate immune responses via both membrane-associated and intracellular receptors, which makes it difficult to unravel the distinct contribution of each component. Most plasma membrane-localized receptors perceive conserved pathogen-associated molecular patterns (PAMPs) or host-cell-derived damage-associated molecular patterns (DAMPs) and activate PAMP-triggered immunity (PTI) or DAMP-triggered immunity (DTI). Plant intracellular immune receptors belong to a family of nucleotide-binding leucine-rich repeat (NB-LRR) proteins, also known as NLRs. NLRs recognize pathogen effectors and activate effector-triggered immunity (ETI), which often leads to accumulation of reactive oxygen species (ROS) and a hypersensitive cell death response (HR). Most plant NLRs carry either coiled-coil (CC) or Toll/interleukin-1 receptor (TIR) N-terminal domains. Both CC and TIR domains are believed to function in signalling upon activation of NLRs, but the detailed mechanisms are unknown. Many CC-NLRs localize at and function in association with the plasma membrane, whereas TIR-NLRs can function in diverse locations, including the nucleus. Regardless of the distinct localization patterns between CC- and TIR-NLRs, their downstream outputs culminate in elevated resistance, but have never been directly compared side-by-side. To study the specific immune outputs generated by ETI, inducible expression tools have been applied (McNellis *et al.*, 1998; Tornero *et al.*, 2002; Allen *et al.*, 2004; Porter *et al.*, 2012).

In Arabidopsis, functionally paired NLRs RRS1-R and RPS4 confer resistance against a soil-borne bacterial pathogen *Ralstonia solanacearum* through the recognition of an effector PopP2 secreted via a Type III secretion system and a hemibiotrophic ascomycetous fungal pathogen *Colletotrichum higginsianum* (Narusaka *et al.*, 2009). They can also confer resistance against the bacterium *Pseudomonas syringae* pv. *tomato* (*Pst*) DC3000 carrying AvrRps4, an effector protein from *Pseudomonas syringae* pv. *pisi*, causing bacterial blight in *Pisum sativum* (pea) (Narusaka *et al.*, 2009; Sohn *et al.*, 2009). Previously, it was reported that residues 135–138 of AvrRps4, lysine–arginine–valine–tyrosine (KRVY), are required for the recognition of AvrRps4 by RRS1 and RPS4 (Sohn *et al.*, 2009). The crystal structure of the C-terminus of AvrRps4 revealed that residue 187 (glutamate; E187) is also required for HR and immunity (Sohn *et al.*, 2012). PopP2 recognition by RRS1 occurs by the integrated WRKY domain at the C-terminus of a resistant allele of RRS1-R [from the Wassilewskija-2 (Ws-2) ecotype of Arabidopsis] but not the susceptible allele of RRS1-S [from the Columbia-0 (Col-0) ecotype of Arabidopsis] (Sarris *et al.*, 2015). Crystal structure information of RRS1 and RPS4 on the TIR domains and the co-crystal structures between the RRS1-R WRKY domain and effector PopP2 have indicated some structural basis for how RRS1/RPS4 have been activated (Williams *et al.*, 2014; Zhang *et al.*, 2017). However, it is still unknown how the protein complex assembles and functions.

Here we report tools for studying the immune complex of RRS1-R and RPS4 *in vivo*. We established a set of transgenic Arabidopsis lines to study RRS1/RPS4-mediated ETI in the absence of pathogens. Using these lines, we show that some, but not all, immune outputs induced by the conditionally expressed AvrRps4 resemble other reported effector-inducible lines.

## Materials and methods

### Plant material and growth conditions

*Arabidopsis thaliana* accessions Ws-2 and Col-0 were used as the wild type in this study. The *eds1-2* mutant used has been described previously (Falk *et al.*, 1999). Seeds were sown on compost, and plants were grown at 21 °C with 10 h under light and 14 h in dark, and at 70% humidity. Tobacco plants were grown at 22 °C with 16 h light/8 h dark, and at 80% constant humidity. The light level is ~180–200 µmol with fluorescent tubes.

### FastRed selection for transgenic Arabidopsis

Seeds harvested from *Agrobacterium*-transformed Arabidopsis were resuspended in 0.1% agarose and exposed under a fluorescence microscope with a DsRed (red fluorescent protein) filter. Seeds with bright red fluorescence are selected as the positive transformants.

### β-Glucuronidase (GUS) staining

*Nicotiana benthamiana* leaves were infiltrated with agrobacteria carrying constructs with the GUS reporter gene expressed under selected Arabidopsis promoters (Supplementary Table S1 at *JXB* online). Leaves were collected at 2 days post-infiltration (dpi), and vacuum-infiltrated with GUS staining buffer [0.1 M sodium phosphate pH 7.0, 10 mM EDTA pH 7.0, 0.5 mM K_3_Fe(CN)_6_, 0.5 mM K_4_Fe(CN)_6_, 0.76 mM 5-bromo-4-chloro-3-indolyl-β-d-glucuronide cyclohexylamine salt or X-Gluc, and 0.04% Triton X-100]. After vacuum infiltration, the leaves were incubated at 37 °C overnight in the dark. The leaves were rinsed with 70% ethanol until the whole leaf de-stained to a clear white.

### Immunoblotting

*Nicotiana benthamiana* leaves were infiltrated with agrobacteria carrying our stacking constructs (Supplementary Table S2). At 2 dpi, the same leaves were infiltrated with either DMSO or 50 µM β-estradiol (E2) diluted in water. Samples were collected at 6 hours post-infiltration (hpi) of DMSO or E2 treatment, and snap-frozen in liquid nitrogen. Proteins were extracted using GTEN buffer (10% glycerol, 25 mM Tris pH 7.5, 1 mM EDTA, 150 mM NaCl) with 10 mM DTT, 1% NP-40, and protease inhibitor cocktail (cOmplete™, EDTA-free; Merck). For Arabidopsis seedlings, seedlings grown for 8 d after germination were treated with DMSO or E2 at the indicated time points and snap-frozen in liquid nitrogen. After centrifugation at 13 000 rpm for 15 min to remove cell debris, the protein concentration of each sample was measured using the Bradford assay (Protein Assay Dye Reagent Concentrate; Bio-Rad). After normalization, extracts were incubated with 3× SDS sample buffer at 95 °C for 5 min; 6% SDS–PAGE gels were used to run the protein samples. After transferring proteins from gels to polyvinylidene fluoride (PVDF) membranes (Merck-Millipore) using the Trans-Blot Turbo System (Bio-Rad), membranes were immunoblotted with horseradish peroxidase (HRP)-conjugated Flag antibodies (Monoclonal ANTI-FLAG^®^ M2-Peroxidase HRP antibody produced in mouse, A5892; Merck-Millipore), HRP-conjugated HA antibodies (12013819001; Merck-Roche), or Phospho-p44/42 mitogen-acivated protein kinase (MAPK; Erk1/2) (Thr202/Tyr204) (D13.14.4E) XP® rabbit monoclonal antibody (4370; Cell Signalling Technology). Anti-rabbit IgG (whole molecule)–peroxidase antibody produced in goat (A0545; Merck-Sigma-Aldrich) was used as secondary antibody following the use of Phospho-p44/42 MAPK antibody.

### Bacterial growth assay

*Pst* strain DC3000 carrying pVSP61 empty vector was grown on selective King’s B (KB) medium plates containing 15% (w/v) agar, 25 µg ml^–1^ rifampicin, and 50 µg ml^–1^ kanamycin for 48 h at 28 °C. Bacteria were harvested from the plates, resuspended in infiltration buffer (10 mM MgCl_2_), and the concentration was adjusted to an optical density of 0.001 at 600 nm [OD_600_=0.001, representing ~5×10^5^ colony-forming units (CFU) ml^–1^]. Bacteria were infiltrated into abaxial surfaces of 5-week-old Arabidopsis leaves with a 1 ml needleless syringe. For quantification, leaf samples were harvested with a 6 mm diameter cork borer (Z165220; Merck-Sigma-Aldrich), resulting in leaf discs with an area of 0.283 cm^2^. Two leaf discs per leaf were harvested as a single sample. For each condition, four samples were collected immediately after infiltration as ‘day 0’ samples to ensure no significant difference introduced by unequal infiltrations, and six samples were collected at 3 dpi as ‘day 3’ samples to compare the bacterial growth between different genotypes, conditions, and treatments. For ‘day 0’, samples were ground in 200 μl of infiltration buffer and spotted (10 μl per spot) on selective KB medium agar plates to grow for 48 h at 28 °C. For ‘day 3’, samples were ground in 200 μl of infiltration buffer, serially diluted (5, 50, 500, 5000, and 50 000 times), and spotted (6 μl per spot) on selective KB medium agar plates to grow for 48 h at 28 °C. The number of colonies (CFU per drop) was monitored and bacterial growth was represented as CFU cm^–2^ of leaf tissue. All results are plotted using ggplot2 in R (Wickham, 2016), and a detailed statistical summary can be found in Supplementary Table S5.

### Hypersensitive cell death response phenotyping in Arabidopsis

*Pseudomonas fluorescens* engineered with a Type III secretion system (Pf0-1 ‘EtHAn’ strains) expressing one of the wild-type or mutant effectors, AvrRps4, AvrRps4^KRVY135-138AAAA^, PopP2, PopP2^C321A^, AvrRpt2, or pVSP61 empty vector were grown on selective KB plates for 24 h at 28 °C (Thomas *et al.*, 2009; Sohn *et al.*, 2014). Bacteria were harvested from the plates, resuspended in infiltration buffer (10 mM MgCl_2_), and the concentration was adjusted to OD_600_=0.2 (10^8^ CFU ml^–1^). The abaxial surfaces of 5-week-old Arabidopsis leaves were hand infiltrated with a 1 ml needleless syringe. Cell death was monitored 24 h after infiltration.

### Electrolyte leakage assay

Either 50 μM E2 or DMSO was hand infiltrated into 5-week-old Arabidopsis leaves with a 1 ml needleless syringe for electrolyte leakage assay. Leaf discs were taken with a 4 mm diameter cork borer from infiltrated leaves. Discs were dried and washed in deionized water for 1 h before being floated on deionized water (15 discs per sample, three samples per biological replicate). Electrolyte leakage was measured as water conductivity with a Pocket Water Quality Meters (LAQUAtwin-EC-33; Horiba) at the indicated time points. All results are plotted using ggplot2 in R (Wickham, 2016), and a detailed statistics summary can be found in Supplementary Table S5.

### Trypan blue staining

Either 50 μM E2 or DMSO was hand infiltrated in 5-week-old Arabidopsis leaves with a 1 ml needleless syringe for trypan blue staining. Six leaves per sample were collected 24 hpi. Leaves were boiled in trypan blue solution (1.25 mg ml^–1^ trypan blue dissolved in 12.5% glycerol, 12.5% phenol, 12.5% lactic acid, and 50% ethanol) in a boiling water bath for 1 min and de-stained by chloral hydrate solution (2.5 g ml^–1^). De-stained leaves were mounted, and pictures were taken under on a Leica M165FC fluorescent stereomicroscope. All images were taken with identical settings at ×2.5 magnification. Scale bars=0.5 mm.

### Reverse transcription–quantitative PCR (RT–qPCR) for measuring relative gene expression

For gene expression analysis, RNA was isolated from 5-week-old Arabidopsis leaves and used for subsequent RT–qPCR analysis. RNA was extracted with a Quick-RNA Plant Kit (R2024; Zymo Research) and treated with RNase-free DNase (4716728001; Merck-Roche). Reverse transcription was carried out using SuperScript IV Reverse Transcriptase (18090050; ThermoFisher Scientific). qPCR was performed using a CFX96 Touch™ Real-Time PCR Detection System. Primers for qPCR analysis of *Isochorismate Synthase1* (*ICS1*), *Pathogenesis-Related1* (*PR1*), *AvrRps4*, and *Elongation Factor 1 Alpha* (*EF1α*) are listed in Supplementary Table S4. Data were analysed using the double delta Ct method (Livak and Schmittgen, 2001). All results are plotted using ggplot2 in R (Wickham, 2016), and a detailed statistical summary can be found in Supplementary Table S5.

### Confocal laser scanning microscopy (CLSM) imaging

Transgenic plant materials were imaged with the Leica DM6000/TCS SP5 confocal microscope (Leica Microsystems) for confirmation of expression of inducible AvrRps4 fused with monomeric yellow–green fluorescent protein, mNeonGreen, or mNeon (Shaner *et al.*, 2013). Roots from 3-week-old Arabidopsis seedlings were sprayed with 50 μM E2 and imaged at 1 d post-spray. Fluorescence of mNeon was excited at 500 nm and detected at between 520 nm and 540 nm. CLSM images of root cells from Arabidopsis seedlings are recorded via the camera. The images were analysed with the Leica application Suite and Fiji software (Schindelin *et al.*, 2012).

### Co-immunoprecipitation (co-IP)

Arabidopsis transgenic seedlings, and the background ecotype Col-0 grown for 7 days after germination (DAG) were treated with 0.1% DMSO or 50 μM E2 for 3 h. Proteins from seedlings were extracted using IP buffer (10% glycerol, 50 mM Tris–HCl pH 6.8, 50 mM KCl, 1 mM EDTA, 5 mM MgCl_2_, 1% NP-40, 10 mM DTT, 1 mM dATP). Crude extract of the seedlings was centrifuged, and supernatants were incubated with anti-HA-conjugated beads (EZviewTM Red Anti-HA Affinity Gel; E6779; Sigma). A small portion of supernatants were taken for input samples. At 2 h after incubation of the extract with beads, the beads were washed three times with IP buffer containing 0.1% NP-40. Proteins bound to beads were eluted by boiling the beads with SDS sample buffer. Immunoblotting of the input and eluted samples was performed as described above.

## Results

### RRS1 overexpression can compromise RPS1/RPS4 function

**Fig. 1.**
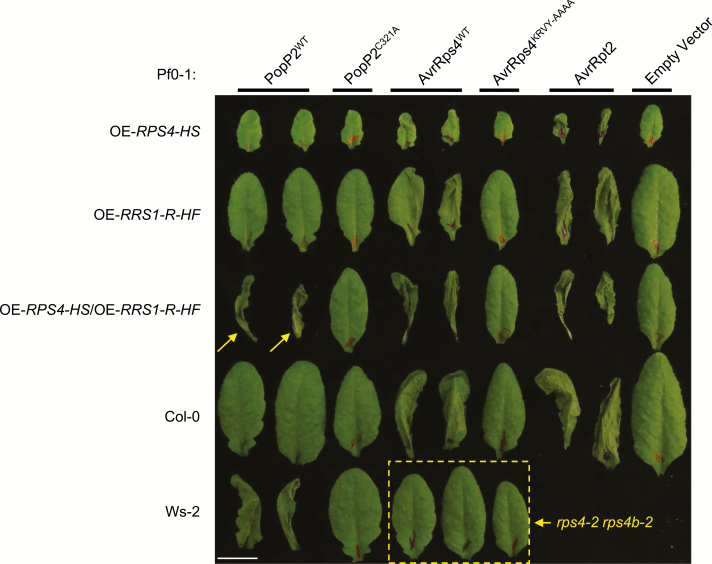
Overexpression of RPS4 and RRS1-R reconstructs the recognition of PopP2 in Col-0. Arabidopsis transgenic lines overexpressing RPS4 (OE-*RPS4-HS*), RRS1-R (OE-*RRS1-R-HF*), or both generated by crossing (OE-*RPS4-HS*/OE-*RRS1-R-HF*) in the Col-0 background, with Col-0 and Ws-2 accessions were tested for hypersensitive response (HR). Five-week-old leaves were infiltrated with *Pseudomonas fluorescens* Pf0-1 strains carrying empty vector (EV), wild-type (WT) AvrRps4, mutant AvrRps4^KRVY-AAAA^, WT PopP2, mutant PopP2^C321A^, and WT AvrRpt2. Leaves were collected 1 day post-infiltration (dpi) for imaging. Scale bar=1 cm. Arrows indicate reconstructed PopP2 recognition of Col-0 background overexpressing RRS1-R and RPS4. The dashed box highlights loss of AvrRps4 recognition in the double mutant *rps4-2 rps4b-2*. Infiltration of EV and AvrRpt2 serves as a negative and positive control of HR, respectively. (This figure is available in colour at *JXB* online.)

Overexpression of *RPS4* leads to autoimmunity and dwarfism under standard growth conditions (see the Materials and methods) (Heidrich *et al.*, 2013). This autoimmunity is both temperature and RRS1 dependent. In contrast, elevated expression of *RRS1-R* from ecotype Ws-2 in Col-0, an ecotype expressing a dominant allele of *RRS1-S*, does not trigger autoimmunity (Huh *et al.*, 2017). Furthermore, high level RRS1-R expression does not confer recognition of effector PopP2 (Fig. 1). Overexpression of *RRS1* in an *RPS4* overexpression line attenuates dwarfism and autoimmunity (Huh *et al.*, 2017). We infiltrated non-pathogenic strains of *P. fluorescens* Pf0-1 engineered with the Type III secretion system from the *Pst* DC3000 strain expressing the effectors PopP2, mutant PopP2^C321A^, AvrRps4, mutant AvrRps4^KRVY-AAAA^, AvrRpt2, and empty vector, respectively (Sohn *et al.*, 2009; Thomas *et al.*, 2009; Saucet *et al.*, 2015). This enabled the assessment of HR activated by individual effectors with their corresponding NLR proteins without artefactual tissue damage from the carrier. The Ws-2 ecotype containing RRS1-R recognizes wild-type PopP2 (PopP2^WT^), whereas the RRS1-S-containing Col-0 ecotype shows no HR with PopP2^WT^ (Fig. 1). Mutant PopP2 (PopP2^C321A^), mutant AvrRps4 (AvrRps4^KRVY-AAAA^), and empty vector served as non-recognition negative controls which do not activate HR (Fig. 1). AvrRpt2 is known to be recognized by CC-NLR RPS2 (Axtell and Staskawicz, 2003; Mackey *et al.*, 2003), and therefore HR was observed in all tested lines. We found that only simultaneously overexpressing *RRS1-R* and *RPS4* can lead to the gain of recognition of PopP2 in the susceptible ecotype Col-0 (Fig. 1). No HR was observed in the *rps4-2 rps4b-2* double mutant when infiltrated with Pf0-1:AvrRps4 (Fig. 1). Thus, we propose that a balanced protein expression of RRS1 and RPS4 is required for both suppressing autoimmunity and functional recognition of the corresponding effectors.

### A survey of leaf-expressed genes reveals promoters for moderate and balanced expression levels of RRS1 and RPS4

Genome-wide expression profiling has revealed numerous genes altered by PTI alone or PTI plus ETI at early time points of RRS1/RPS4-mediated immune activation (Sohn *et al.*, 2014). This analysis also enabled the discovery of genes that are moderately and constitutively expressed without changing their transcript abundance during immune activation. In plants, gene expression patterns and levels are usually specified by their promoters. Based on the endogenous expression relative transcript abundance in the ‘stable gene set’, we selected six promoters with ‘moderate’ expression (Supplementary Table S1). We define the ‘moderate’ expression based on two criteria: (i) the gene transcript abundance with those promoters is at least 100 times more than the endogenous transcript abundance of *RRS1* and *RPS4*; and (ii) the gene transcript abundance with those promoters is lower than that with the 35S promoter. The selected genes encode proteins that are involved in essential biological processes that we expect to be expressed in most mesophyll cells, including a δ-tonoplast intrinsic protein (our name: At1, locus identifier AT3G16240, protein symbol name TIP2-1), a ribosomal protein S16 (At2, AT4G34620, RPS16-1), a cysteine synthase isomer CysC1 (At3, AT3G61440, CYSC1), a PSII subunit Q (At4, AT4G21280, PSBQ1), a xyloglucan endotransglucosylase/hydrolase 6 (At5, AT5G65730, XTH6), and a ubiquitin-like protein 5 (At6, AT5G42300, UBL5) (Supplementary Table S1).

To test the strength of the selected Arabidopsis promoters (pAt1–pAt6) for driving gene expression *in planta*, constructs were designed and generated to express GUS (pAt:GUS). *Agrobacterium* strains carrying each pAt:GUS construct were infiltrated in *N. benthamiana* leaves with the infiltration buffer as negative control and GUS expressed under the *Cauliflower mosaic virus* (CaMV) 35S promoter (35S:GUS) as positive control. GUS expressed under pAt4 shows a similar level of activity to that with 35S, whereas GUS activities detected from other pAt promoters are significantly weaker (Supplementary Fig. S1).

### A T-DNA construct expresses RPS4, RRS1, and inducible AvrRps4

**Fig. 2.**
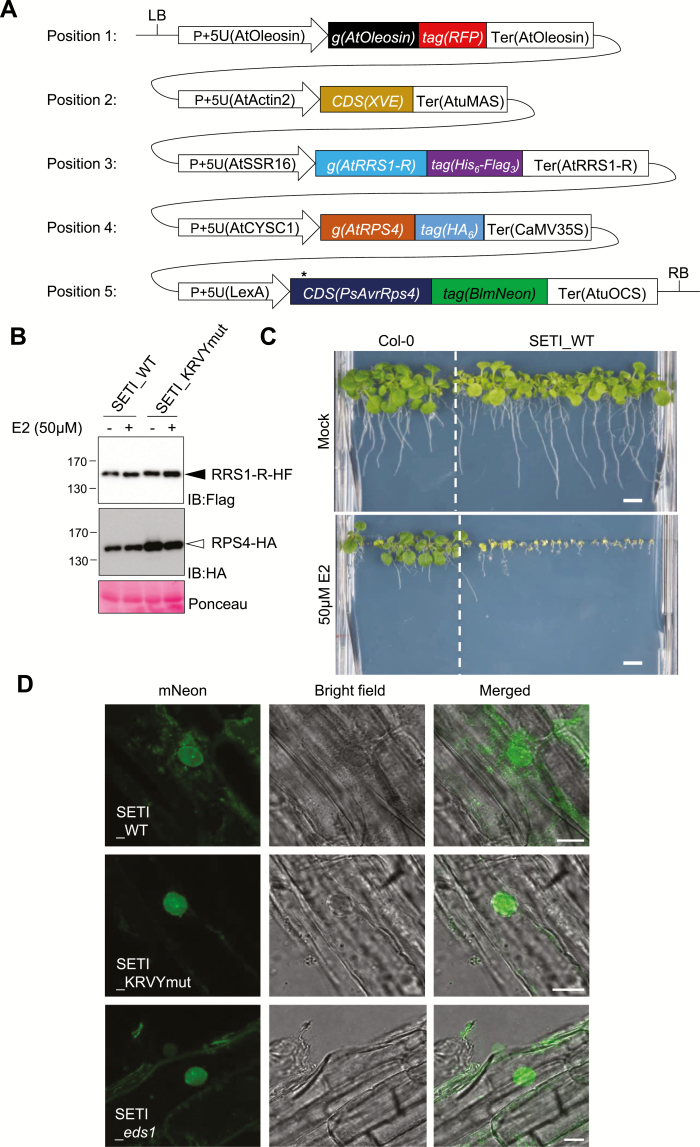
Single T-DNA expresses RRS1-R-HF, RPS4-HA, and inducible wild-type AvrRps4 or AvrRps4 mutant variants. (A) Illustrative layout of the Super-ETI (SETI) construct. There are five individual expression units or Golden Gate Level 2 positional components listed, which are indicated as positions 1–5. Position 1, expression unit of the FastRed selection marker (Shimada *et al.*, 2010). Positions 2 and 5, chimeric transactivator XVE (LexA-VP16-ER) and the corresponding LexA-inducible system to express AvrRps4 or its mutant variants under the control of β-estradiol (E2) treatment. Positions 3 and 4, full-length RRS1-R and RPS4 proteins with epitope tags His_6_-Flag_3_ and HA_6_, respectively. All individual units used for construct assembly can be found in Supplementary Tables S2 and S3. (B) Protein accumulation of RRS1-R-HF (IB:Flag, black arrowhead) and RPS4-HA (IB:HA, white arrowhead) of SETI lines expressing AvrRps4 (SETI_WT) or mutant AvrRps4 KRVY-AAAA (SETI_KRVYmut). Seedlings were grown in liquid culture and induced with 50 μM E2 for 2 h at 7 days after germination (DAG). Ponceau staining of Rubisco large subunits was used as a loading control. (C) Seedling phenotype of the SETI Arabidopsis transgenic line at 14 DAG in GM medium containing mock (0.1% DMSO) or 50 μM E2. Col-0 was sown as control for the effect of E2 on seedling growth. Scale bar=0.5 cm. (D) Confocal images of SETI_WT, SETI_KRVYmut, SETI_*eds1* root cells expressing AvrRps4–mNeon, and AvrRps4^KRVY-AAAA^–mNeon induced by 50 μM E2 for 24 h. The mNeon channel shows nucleocytoplasmic localization of AvrRps4–mNeon and AvrRps4^KRVY-AAAA^–mNeon. Bright field channel and a merged image of mNeon and the bright field channel are shown together. Scale bars=10 μm. (This figure is available in colour at *JXB* online.)

We designed a binary vector to reconstruct the effector ligand AvrRps4 and its receptors RRS1 and RPS4, using the Golden Gate Modular Cloning Toolbox (Fig. 2A) (Engler *et al.*, 2014). We chose moderate and balanced promoters pAt2 and pAt3 from our promoter survey experiment for expressing RRS1 and RPS4, respectively. We have also cloned the *RRS1-R* full-length coding sequence (CDS) from Ws-2 and the *RPS4* full-length CDS from Col-0 for the expression of RRS1-R and RPS4 proteins. We chose synthetic C-terminal fusion epitope tags His_6_-TEV-FLAG_3_ (HF) and HA_6_ for detecting RRS1 and RPS4 protein expression, respectively (Fig. 2A; Supplementary Table S2) (Gauss *et al.*, 2005; Soleimani *et al.*, 2013). We have used an E2-inducible system for AvrRps4 expression (Zuo *et al.*, 2000). We named this multigene stacking binary construct ‘Super ETI’, or SETI. We have also generated constructs inducing mutant AvrRps4^KRVY-AAAA^ or mutant AvrRps4^E187A^ as negative controls, and named them SETI_KRVYmut and SETI_E187A, respectively. SETI_KRVYmut and SETI_E187A can induce the expression of mutant AvrRps4 alleles, but no induction of immunity because these two mutant AvrRps4 alleles cannot be recognized by RRS1 and RPS4 (Sohn *et al.*, 2009, 2014). All restriction enzyme sites for *Bsa*I and *Bpi*I in modules for promoters, CDSs for genes or epitope tags, and the terminators were eliminated (Fig. 2A; Supplementary Table S4). More detailed information on the cloning can be found in Supplementary Table S3. To verify the SETI construct, we used a transient expression system in *N. benthamiana* by infiltrating agrobacteria that deliver the SETI T-DNA. Protein accumulation of RRS1-R-HF and RPS4-HA was detected (Supplementary Fig. S2).

### The single-locus lines carrying the SETI T-DNA show inducible growth arrest

We generated transgenic Arabidopsis lines using the SETI, SETI_KRVYmut, and SETI_E187A construct expressing AvrRps4 (SETI_WT), AvrRps4^KRVY-AAAA^, and AvrRps4^E187A^, respectively. With the FastRed selection module, we have selected ~20 positive SETI_WT T_1_ lines. The seedlings from the T_2_ generation of three T_1_ lines were further tested for response to E2 treatment (see the Materials and methods; Supplementary Table S2). On E2-containing growth medium, SETI_WT transgenic lines display severe growth arrest (Supplementary Fig. S3). We selected one of the lines (T1-#8_T2-#4; SETI_WT) for subsequent experiments (Fig. 2C; Supplementary Fig. S3). We confirmed the protein expression of RRS1-R-HF and RPS4-HA (Fig. 2B). We also tested the expression of inducible AvrRps4–mNeon under a fluorescence microscope upon treatment with E2. mNeonGreen signal was detected at 24 h post-spray on transgenic seedlings, consistent with the mRNA accumulation of *AvrRps4* at 4 h post E2 infiltration in leaves (Fig. 2D; Supplementary Fig. S2C).

### RRS1-R and RPS4 form pre-activation complexes in Arabidopsis

**Fig. 3.**
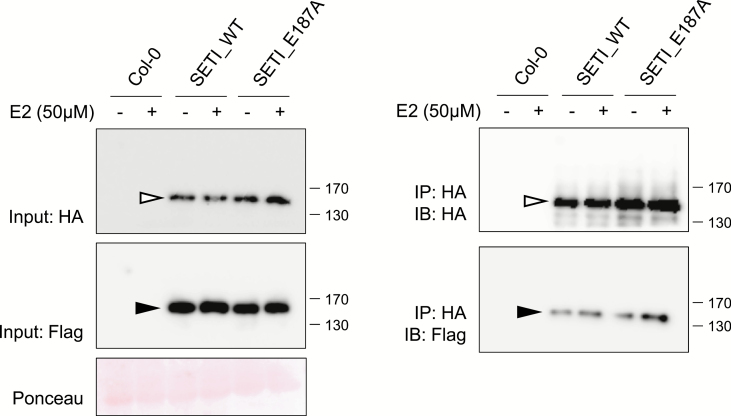
RRS1-R and RPS4 interact *in vivo*. Co-immunoprecipitation of RRS1-R-HF with RPS4-HA. Col-0, SETI_WT, and SETI_E187A seedlings at 7 DAG were treated with 50 μM E2 for 3 h. Crude extracts were centrifuged and RPS4-HA proteins were immunoprecipitated with anti-HA-conjugated beads. Immunoprecipitation of RPS4-HA, and co-immunoprecipitation of RRS1-R-HF were determined by immunoblot analysis with HA (IB:HA) or Flag (IB:Flag). Ponceau staining indicates equal loading of the input samples. RRS1-R-HF (black arrowhead) and RPS4-HA (white arrowhead) are indicated.

The SETI lines enable detection of epitope-tagged RRS1-R and RPS4 (Fig. 2B). We investigated *in vivo* interaction of tagged RRS1-R and RPS4 by co-IP with SETI_WT and SETI_E187A seedling extracts with or without E2 induction. When RPS4-HA was immunoprecipitated using HA beads, we found that RRS1-R and RPS4 stay in association with each other both before and 3 h after the induction of *AvrRps4* expression (Fig. 3). There were no significant differences of RRS1-R and RPS4 association upon *AvrRps4* induction. Induction of AvrRps4 E187A also had no effect on RRS1-R and RPS4 association. While all previous studies on interactions of RRS1-R and RPS4 only used the *N. benthamiana* transient expression system (Huh *et al.*, 2017), generation of the SETI lines enabled the detection of RRS1-R and RPS4 interaction in their native system in Arabidopsis.

### Some but not all defence responses are induced by E2 in SETI lines

**Fig. 4.**
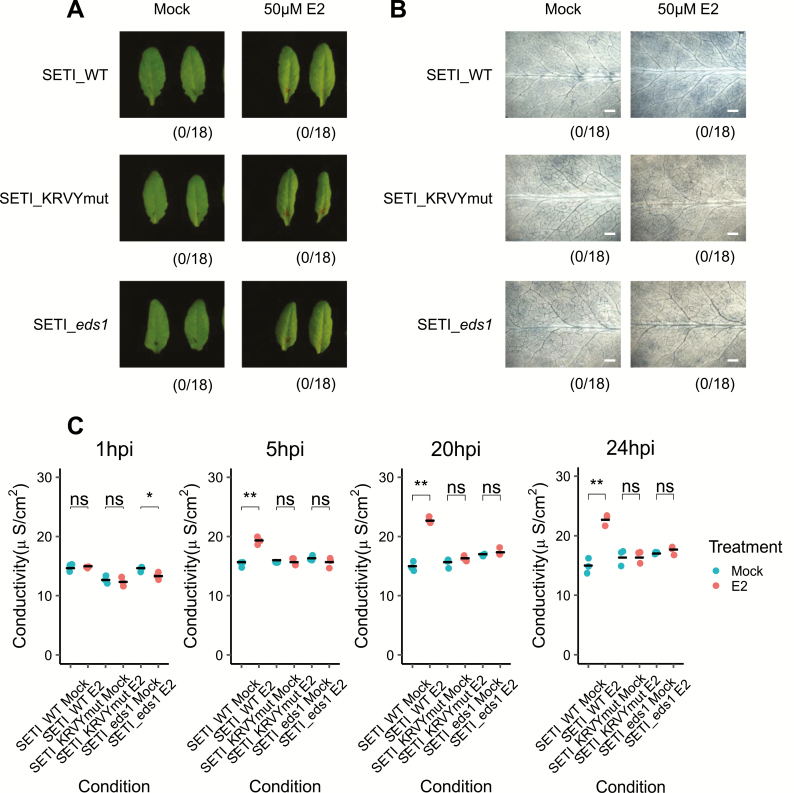
Induced expression of AvrRps4 in Arabidopsis causes microscopic but not macroscopic cell death. (A) HR phenotype assay in Arabidopsis. Five-week-old SETI_WT, SETI_KRVYmut, and SETI_*eds1* leaves were mock infiltrated (1% DMSO) or infiltrated with 50 μM E2. Images were taken at 1 dpi. Numbers indicate the number of leaves displaying cell death from the total number of infiltrated leaves (18 for each genotype and treatment). (B) Trypan blue staining. Five-week-old SETI_WT, SETI_KRVYmut, and SETI_*eds1* were mock infiltrated (1% DMSO) or infiltrated with 50 μM E2. Leaves were stained with trypan blue solution at 1 dpi. After destaining, leaves were imaged using a stereoscopic microscope. Scale bar=0.5 mm. (C) Electrolyte leakage assay. Five-week old SETI_WT, SETI_KRVYmut, and SETI_*eds1* leaves were mock infiltrated (1% DMSO) or infiltrated with 50 μM E2. Fifteen leaf discs were collected for each data point. Conductivity was measured at 1, 5, 20, and 24 hours post-infiltration (hpi). Each data point represents one technical replicate, and three technical replicates are included per treatment and genotype for one biological replicate. The black line represents the mean of the technical replicates. This experiment was repeated three times independently with similar results (Supplementary Fig. S2). Significant differences relative to the mock treatment in each genotype were calculated with *t*-test, and the *P*-values are indicated as ns (non-significant), *P*>0.05; *P<0.05; **P<0.01; ***P<0.001. (This figure is available in colour at *JXB* online.)

The induced expression of multiple effectors, such as AvrRpt2, AvrRpm1, and ATR13, can induce cell death or specific macroscopic HR in Arabidopsis leaves (McNellis *et al.*, 1998; Tornero *et al.*, 2002; Allen *et al.*, 2004). We therefore tested whether induced expression of AvrRps4 can trigger macroscopic HR in Arabidopsis. We used SETI_*eds1* as control, in which SETI_WT was crossed with the mutant *eds1*. EDS1 is a downstream genetic component of TIR-NLR-mediated ETI (Aarts *et al.*, 1998; Falk *et al.*, 1999). As seen in Fig. 4A, no HR can be observed after AvrRps4 expression is induced in the SETI leaves. However, only the expression of AvrRps4 but not that of AvrRps4_KRVYmut leads to electrolyte leakage (Fig. 4C). We also observed slightly stronger trypan blue stains in the SETI leaves treated with E2 compared with mock treatment; suggesting that the expression of AvrRps4 causes microscopic or weak but not macroscopic or strong HR in contrast to other known inducible effector-expressing lines (Fig. 4B).

**Fig. 5.**
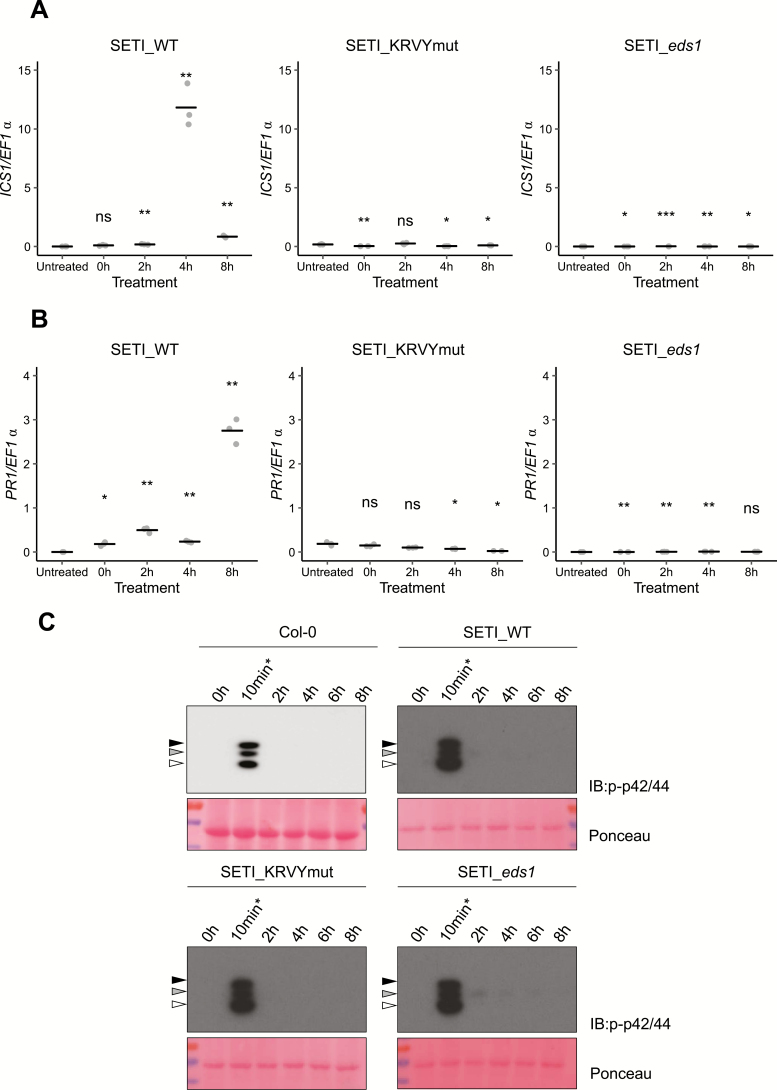
Induced expression of AvrRps4 in Arabidopsis leads to *ICS1* and *PR1* expression, but not MAPK activation. (A and B) *ICS1* (A) and *PR1* (B) expression after induction with E2 for 2, 4, and 8 h in SETI (left panel), SETI_KRVYmut (middle panel), and SETI_eds1 (right panel) leaf samples. Five-week-old SETI and SETI_KRVYmut leaves were infiltrated with 50 μM E2. Samples were collected at 0, 2, 4, and 8 hpi for RNA extraction and subsequent qPCR. The expression level is presented as relative to *EF1α* expression. Each data point represents one technical replicate. The black line represents the mean of the technical replicates. This experiment was repeated three times independently with similar results. Significant differences relative to the untreated samples were calculated with *t*-test, and the *P*-values are indicated as ns (non-significant), *P*>0.05; *P<0.05; **P<0.01; ***P<0.001. (C) Activation of MAPKs in Col-0, SETI_WT, SETI_KRVYmut, and SETI_*eds1* seedlings by E2 induction of effector AvrRps4 or mutant AvrRps4^KRVY-AAAA^. Seedlings grown in liquid culture at 7 DAG were treated with 50 μM E2 for the indicated times (0, 2, 4, 6, or 8 h) and samples were collected. Col-0, SETI_WT, SETI_KRVYmut, and SETI_*eds1* seedlings treated with 100 nM flg22 for 10 min (10 min*) were used as positive control. Proteins were extracted from these seedlings and phosphorylated MAPKs were detected using p-p42/44 antibodies. Arrowheads indicate phosphorylated MAPKs (black, pMPK6; grey, pMPK3; white, pMPK4/11). Ponceau staining was used as loading control. (This figure is available in colour at *JXB* online.)

Salicylic acid induction is another hallmark of ETI (Castel *et al.*, 2019). Enzymes such as isochorismate synthase 1 (ICS1), enhanced disease susceptibility 5 (EDS5), and AvrPphB susceptible 3 (PBS3) are involved in the biosynthesis of salicylic acid (Rekhter *et al.*, 2019; Torrens-Spence *et al.*, 2019), and the expression of these genes is also highly induced during ETI (Sohn *et al.*, 2014). The expression of *ICS1* after *AvrRps4* induction was tested by quantitative real-time PCR. *ICS1* was highly induced 4 h after the induction of *AvrRps4* by E2 but not in the negative controls of SETI_KRVYmut or SETI_*eds1* (Fig. 5A). In contrast, the *Pathogenesis-Related protein 1* (*PR1*) gene was highly induced only 8 h after the induction of *AvrRps4* (Fig. 5B). This shows that ETI triggered by RRS1/RPS4 is sufficient for the induction of *ICS1* and the biosynthesis of salicylic acid, which subsequently leads to expression of *PR1*.

Activation of MAPKs by PTI has been reported in many cases, and happens within a few minutes of the activation of PTI. However, the activation of MAPKs by ETI is slower and lasts longer than PTI-induced MAPK activation (Tsuda *et al.*, 2013). We tested whether the induced expression of *AvrRps4* can lead to MAPK activation in SETI_WT and control lines Col-0, SETI_KRVYmut, and SETI_*eds1*. Treatment with flg22 for 10 min triggered phosphorylation of MAPKs (Fig. 5C). However, in contrast to AvrRpt2-inducible transgenic Arabidopsis plants (Tsuda *et al.*, 2013), induced expression of AvrRps4 does not activate MAPKs (Fig. 5C).

**Fig. 6.**
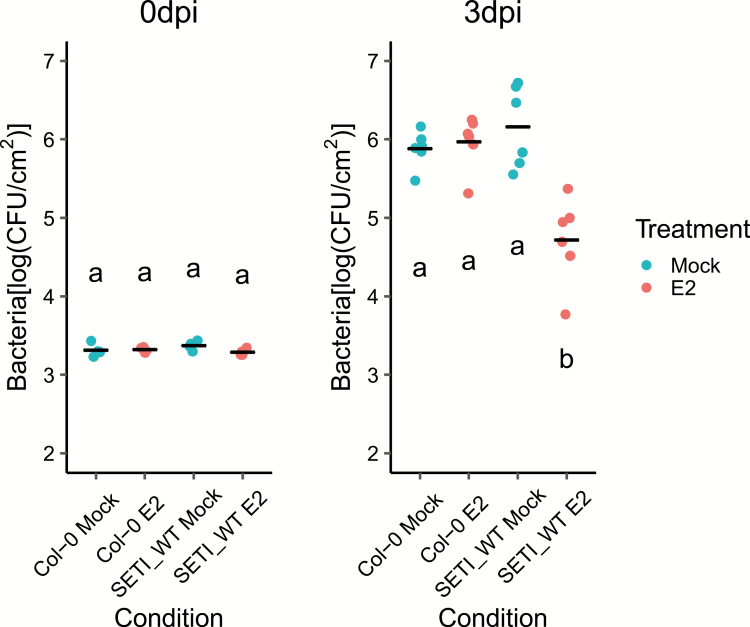
Effector-triggered immunity triggered by the expression of AvrRps4 leads to resistance against *Pseudomonas syringae* pv. *tomato* strain DC3000. Five-week-old SETI_WT and Col-0 leaves were mock infiltrated (1% DMSO) or infiltrated with 50 μM E2. At 1 dpi, leaves were inoculated with *Pst* DC3000 (OD_600_=0.001). Bacteria in the leaves were then quantified as colony-forming units (CFU) at 0 and 3 dpi. Each data point represents two leaves collected from one individual plant. Samples from four individual plants were collected for 0 dpi and samples from six individual plants were collected for 3 dpi. The black line represents the mean of the technical replicates. This experiment was repeated three times independently with similar results. Biological significance of the values was determined by one-way ANOVA followed by post-hoc Tukey HSD analysis. Letters above the data points indicate significant differences (*P*<0.05). (This figure is available in colour at *JXB* online.)

We further tested if the induction of ETI would elevate resistance. We infiltrated the leaves with E2 or mock solution 1 d before we infiltrated plants with *Pst* DC3000 (see the Materials and methods). SETI_WT plants pre-treated with E2 are more resistant to the bacteria than those mock pre-treated, while there was no significant difference between E2 and mock pre-treatment in Col-0 (Fig. 6).

## Discussion

To facilitate studying the functional complex of RRS1 and RPS4 *in vivo*, we generated an expression construct of E2-inducible AvrRps4 stacked with epitope-tagged RRS1 and RPS4. To achieve balanced expression levels higher than endogenous expression of *RRS1* and *RPS4*, we surveyed constitutively expressed gene promoters. Here, we report six new and tested promoter modules that are compatible with the Golden Gate Modular Cloning Toolbox. We used two of the promoters to express *RRS1* and *RPS4*, and we avoided autoimmunity induced by excessive expression of *RPS4* or non-recognition of PopP2 caused by excessive expression of *RRS1-R*. We were also able to generate inducible AvrRps4 expression to activate RRS1/RPS4-mediated ETI under the control of E2 treatment. We thus were able to stack genes for inducible expression of a pathogen effector and its NLR receptors in one construct. In addition, with the epitope tags, we are able to monitor effector-dependent changes in the NLR proteins without interference from using a pathogen effector delivery system. We could thus express any effectors or pathogen ligands that will trigger immunity in plant cells with the E2-inducible module, and their immune receptors using the same gene stacking strategy.

There are multiple advantages to enabling investigation of ETI without the complication of co-activating PTI. First, we could test the contribution of other genes to ETI activation by introducing mutants into the SETI background, either using conventional crossing or using genome editing such as CRISPR/Cas9 [clustered regularly interspaced short palindromic repeats (CRISPR)/CRISPR-associated protein 9]. These lines can also help in investigating downstream signalling from plant NLRs. Multiple forward genetic screens have been conducted, but few novel components have been found, and most mutations are in either the NLRs or regulatory elements rather than signalling components (van Wersch *et al.*, 2016). Another plausible explanation is that the signalling path downstream of plant NLRs is very short, but this is debatable because several significant steps are required for immunity. EDS1, PAD4, and SAG101 are required for TIR–NLR signalling (Falk *et al.*, 1999; Gantner *et al.*, 2019). NRC family proteins in Solanaceae species are required for many NLRs, NRG1/ADR1s in Arabidopsis are required for TIR-NLRs, and ADR1s are required for some CC-NLRs (Bonardi *et al.*, 2011; Dong *et al.*, 2016; Wu *et al.*, 2017; Castel *et al.*, 2019; Wu *et al.*, 2019). NRG1s and ADR1s seem to function downstream of EDS1 and may function distinctly with SAG101 and PAD4, respectively (Lapin *et al.*, 2019). SETI lines carry heterologously expressed RRS1-R/RPS4 and also endogenous RRS1-S/RPS4 and RRS1B/RPS4B, which together provide three redundant copies of NLR pairs that can recognize AvrRps4. In theory, in an ethyl methanesulfonate (EMS) mutagenesis forward genetic screen to identify suppressors of immunity induced by AvrRps4, there should be a reduced background of mutations in the receptor(s), improving prospects to reveal mutations in genes that are functionally important in NLR signalling and regulation.

With SETI, we are able to assess the pure ETI response mediated by the TIR-NLRs, RRS1 and RPS4. E2 induction provoked rapid transcriptional changes in activation of defence genes and also ion leakage. AvrRps4-induced ETI-enhanced resistance against bacterial pathogens; but neither MAPK activation nor macroscopic HR, in contrast to other inducible ETI examples (Tornero *et al.*, 2002; Tsuda *et al.*, 2013). This indicates that outputs of plant NLRs might differ. Both TIR and CC domains alone are sufficient to activate plant immunity. However, whether they signal through similar or different downstream components is still unknown.

In diverse multicellular eukaryotes, immune complexes are assembled into oligomeric complexes to signal downstream. The mammalian inflammasome, assembled in response to bacterial peptide recognition by NAIP proteins and subsequent activation and binding of NLRC4 proteins, is a classic example (Zhang *et al.*, 2015). The plant CC-NLR ZAR1 forms an effector-dependent resistosome, which is a pentamer of ZAR1 assembled together with cofactors PBL2 and RKS1 (Wang *et al.*, 2019). The structure of TIR domains implies that activation might require the disassociation of the RRS1 and RPS4 TIR domains and the oligomerization of RPS4 TIR domains (Williams *et al.*, 2014). In SETI lines, RRS1 and RPS4 form a pre-activation complex in the absence of pathogen effector. However, co-IP data cannot distinguish the ratio with which RRS1 and RPS4 bind to each other. It will be interesting to check via various non-denaturing methods if RRS1-R and RPS4 form a dimer or a higher order oligomer *in vivo*, or whether there is a conformational change in the complex upon effector recognition. Furthermore, with the SETI lines generated in this study, we can ask what other cofactors are required for the activation of RRS1-R and RPS4 under native conditions.

The availability of SETI lines will also enable us to study how PTI and ETI interact with each other, especially in the context of RRS1- and RPS4-mediated immunity. Some models have been proposed in discussing this topic (Tsuda *et al.*, 2009; Cui *et al.*, 2015). From the zig–zag model, PTI and ETI have different thresholds for activation of immunity (Jones and Dangl, 2006). With the SETI line, we could specifically ask how PTI and ETI can physically influence each other. A lot of evidence shows that the PTI receptors PRRs usually have very specific post-translational modification (PTM) events at early time points; there is also some evidence showing that ETI can activate somewhat overlapping but different PTMs on immune-related proteins (Withers and Dong, 2017; Kadota *et al.*, 2019). It will be interesting to know how the activation of RRS1/PRS4 leads to the changes of PTMs and how those changes contribute to the robustness of immunity. In addition, transcriptional changes are not the only process reported as the early changes of ETI, but also changes in translation (Meteignier *et al.*, 2017; Yoo *et al.*, 2020). Work using inducible AvrRpm1 or AvrRpt2 reveals interesting observations on the trade-off between defence and growth, and the specific regulatory element in the genome (Meteignier *et al.*, 2017; Yoo *et al.*, 2020). Both effectors are recognized by CC-type NLRs, so it will be interesting to know what changes in translation will be induced by TIR-NLRs using the SETI line. One can also use proteomics tools to generate complex information using inducible SETI to fish for ETI-specific interaction networks.

Recently it has been shown that plant NLRs can also form higher order protein complexes, similar to the inflammasome in the mammalian immune system. However, it is unknown if all plant NLRs form the same kind of complex or use the same mechanism to activate defence. It was noted that NLRs have evolved to partner with other NLRs to function genetically, but whether this model is also true biochemically is still unknown (Adachi *et al.*, 2019). Unlike ZAR1, RRS1 and RPS4 require each other to function, and they localize and function exclusively in the nuclei but not the cell membrane, so it will be interesting to compare them once the cryo-EM structure of the RRS1 and RPS4 complex is resolved. The SETI line could be a very good toolkit for mutagenesis to verify the function based on the structural information.

We have observed that the activation of ETI alone in the absence of pathogens is sufficient to prime the resistance against bacterial pathogens in Arabidopsis (Fig. 6). Previously, we have reported that a group of up-regulated genes at the early time point of activation of RRS1-R/RPS4 are related to the salicylic acid pathway, so it will be interesting to determine whether the elevated or primed resistance against bacteria induced in SETI lines is due to the activation of the salicylate pathway (Sohn *et al.*, 2014).

Another major question regarding the signalling pathways is that SAG101 and PAD4 seem to be redundant but functionally equivalent to EDS1 (Wagner *et al.*, 2013; Lapin *et al.*, 2019). They also have been shown to be genetically linked to helper NLRs, NRG1s and/or ADR1s, to function (Castel *et al.*, 2019; Wu *et al.*, 2019). Using the SETI line, one can test their function more specifically in ETI in the absence of PTI and interference by many other unwanted pathogens.

## Supplementary data

Supplementary data are available at *JXB* online.

Table S1. Information on synthetic promoters used in this study.

Table S2. Golden Gate stacking construct of R genes and effectors.

Table S3. Golden Gate cloning modules used in this work.

Table S4. Primers used in this study.

Table S5. Statistical analysis results.

Fig. S1. GUS-staining activity of synthetic promoters in *N. benthamiana*.

Fig. S2. Transient expression of Super-ETI (SETI) constructs in *N. benthamiana*.

Fig. S3. SETI T_2_ lines grown under E2 treatment.

erz571_suppl_Supplementary-Tables-S1-S5_Figures-S1-S3

## References

[CIT0001] Aarts N, Metz M, Holub E, Staskawicz BJ, Daniels MJ, Parker JE 1998 Different requirements for EDS1 and NDR1 by disease resistance genes define at least two R gene-mediated signaling pathways in Arabidopsis. Proceedings of the National Academy of Sciences, USA 95, 10306–10311. 10.1073/pnas.95.17.10306 PMC21504 9707643

[CIT0002] Adachi H, Derevnina L, Kamoun S 2019 NLR singletons, pairs, and networks: evolution, assembly, and regulation of the intracellular immunoreceptor circuitry of plants. Current Opinion in Plant Biology 50, 121–131. 31154077 10.1016/j.pbi.2019.04.007

[CIT0003] Allen RL, Bittner-Eddy PD, Grenville-Briggs LJ, Meitz JC, Rehmany AP, Rose LE, Beynon JL 2004 Host–parasite coevolutionary conflict between Arabidopsis and downy mildew. Science 306, 1957–1960. 15591208 10.1126/science.1104022

[CIT0004] Axtell MJ, Staskawicz BJ 2003 Initiation of RPS2-specified disease resistance in Arabidopsis is coupled to the AvrRpt2-directed elimination of RIN4. Cell 112, 369–377. 12581526 10.1016/s0092-8674(03)00036-9

[CIT0005] Bonardi V, Tang S, Stallmann A, Roberts M, Cherkis K, Dangl JL 2011 Expanded functions for a family of plant intracellular immune receptors beyond specific recognition of pathogen effectors. Proceedings of the National Academy of Sciences, USA 108, 16463–16468. 10.1073/pnas.1113726108 PMC3182704 21911370

[CIT0006] Castel B, Ngou PM, Cevik V, Redkar A, Kim DS, Yang Y, Ding P, Jones JDG 2019 Diverse NLR immune receptors activate defence via the RPW8-NLR NRG1. New Phytologist 222, 966–980. 30582759 10.1111/nph.15659

[CIT0007] Cui H, Tsuda K, Parker JE 2015 Effector-triggered immunity: from pathogen perception to robust defense. Annual Review of Plant Biology 66, 487–511. 10.1146/annurev-arplant-050213-040012 25494461

[CIT0008] Dong OX, Tong M, Bonardi V, El Kasmi F, Woloshen V, Wünsch LK, Dangl JL, Li X 2016 TNL-mediated immunity in Arabidopsis requires complex regulation of the redundant ADR1 gene family. New Phytologist 210, 960–973. 27074399 10.1111/nph.13821

[CIT0009] Engler C, Youles M, Gruetzner R, Ehnert TM, Werner S, Jones JD, Patron NJ, Marillonnet S 2014 A golden gate modular cloning toolbox for plants. ACS Synthetic Biology 3, 839–843. 24933124 10.1021/sb4001504

[CIT0010] Falk A, Feys BJ, Frost LN, Jones JD, Daniels MJ, Parker JE 1999 EDS1, an essential component of R gene-mediated disease resistance in Arabidopsis has homology to eukaryotic lipases. Proceedings of the National Academy of Sciences, USA 96, 3292–3297. 10.1073/pnas.96.6.3292 PMC15935 10077677

[CIT0011] Gantner J, Ordon J, Kretschmer C, Guerois R, Stuttmann J 2019 An EDS1–SAG101 complex is essential for TNL-mediated immunity in *Nicotiana benthamiana*. The Plant Cell 31, 2456–2474. 31266900 10.1105/tpc.19.00099 PMC6790086

[CIT0012] Gauss R, Trautwein M, Sommer T, Spang A 2005 New modules for the repeated internal and N-terminal epitope tagging of genes in *Saccharomyces cerevisiae*. Yeast 22, 1–12. 15565729 10.1002/yea.1187

[CIT0013] Heidrich K, Tsuda K, Blanvillain-Baufumé S, Wirthmueller L, Bautor J, Parker JE 2013 Arabidopsis TNL-WRKY domain receptor RRS1 contributes to temperature-conditioned RPS4 auto-immunity. Frontiers in Plant Science 4, 403. 24146667 10.3389/fpls.2013.00403 PMC3797954

[CIT0014] Huh SU, Cevik V, Ding P, Duxbury Z, Ma Y, Tomlinson L, Sarris PF, Jones JDG 2017 Protein–protein interactions in the RPS4/RRS1 immune receptor complex. PLoS Pathogens 13, e1006376. 28475615 10.1371/journal.ppat.1006376 PMC5435354

[CIT0015] Jones JD, Dangl JL 2006 The plant immune system. Nature 444, 323–329. 17108957 10.1038/nature05286

[CIT0016] Kadota Y, Liebrand TWH, Goto Y, et al 2019 Quantitative phosphoproteomic analysis reveals common regulatory mechanisms between effector- and PAMP-triggered immunity in plants. New Phytologist 221, 2160–2175. 30300945 10.1111/nph.15523 PMC6367033

[CIT0017] Lapin D, Kovacova V, Sun X, et al 2019 A coevolved EDS1–SAG101–NRG1 module mediates cell death signaling by TIR-domain immune receptors. The Plant Cell 31, 2430–2455. 31311833 10.1105/tpc.19.00118 PMC6790079

[CIT0018] Livak KJ, Schmittgen TD 2001 Analysis of relative gene expression data using real-time quantitative PCR and the 2(-Delta Delta C(T)) method. Methods 25, 402–408. 11846609 10.1006/meth.2001.1262

[CIT0019] Mackey D, Belkhadir Y, Alonso JM, Ecker JR, Dangl JL 2003 Arabidopsis RIN4 is a target of the type III virulence effector AvrRpt2 and modulates RPS2-mediated resistance. Cell 112, 379–389. 12581527 10.1016/s0092-8674(03)00040-0

[CIT0020] McNellis TW, Mudgett MB, Li K, Aoyama T, Horvath D, Chua NH, Staskawicz BJ 1998 Glucocorticoid-inducible expression of a bacterial avirulence gene in transgenic Arabidopsis induces hypersensitive cell death. The Plant Journal 14, 247–257. 9628020 10.1046/j.1365-313x.1998.00106.x

[CIT0021] Meteignier LV, El Oirdi M, Cohen M, Barff T, Matteau D, Lucier JF, Rodrigue S, Jacques PE, Yoshioka K, Moffett P 2017 Translatome analysis of an NB-LRR immune response identifies important contributors to plant immunity in Arabidopsis. Journal of Experimental Botany 68, 2333–2344. 28369573 10.1093/jxb/erx078

[CIT0022] Narusaka M, Shirasu K, Noutoshi Y, Kubo Y, Shiraishi T, Iwabuchi M, Narusaka Y 2009 RRS1 and RPS4 provide a dual Resistance-gene system against fungal and bacterial pathogens. The Plant Journal 60, 218–226. 19519800 10.1111/j.1365-313X.2009.03949.x

[CIT0023] Porter K, Shimono M, Tian M, Day B 2012 Arabidopsis actin-depolymerizing factor-4 links pathogen perception, defense activation and transcription to cytoskeletal dynamics. PLoS Pathogens 8, e1003006. 23144618 10.1371/journal.ppat.1003006 PMC3493479

[CIT0023a] Rekhter D, Lüdke D, Ding Y, Feussner K, Zienkiewicz K, Lipka V, Wiermer M, Zhang Y, Feussner I 2019 Isochorismate-derived biosynthesis of the plant stress hormone salicylic acid. Science 365, 498–502. 31371615 10.1126/science.aaw1720

[CIT0024] Sarris PF, Duxbury Z, Huh SU, et al 2015 A plant immune receptor detects pathogen effectors that target WRKY transcription factors. Cell 161, 1089–1100. 26000484 10.1016/j.cell.2015.04.024

[CIT0025] Saucet SB, Ma Y, Sarris PF, Furzer OJ, Sohn KH, Jones JD 2015 Two linked pairs of Arabidopsis TNL resistance genes independently confer recognition of bacterial effector AvrRps4. Nature Communications 6, 6338. 10.1038/ncomms7338 25744164

[CIT0026] Schindelin J, Arganda-Carreras I, Frise E, et al 2012 Fiji: an open-source platform for biological-image analysis. Nature Methods 9, 676–682. 22743772 10.1038/nmeth.2019 PMC3855844

[CIT0027] Shaner NC, Lambert GG, Chammas A, et al 2013 A bright monomeric green fluorescent protein derived from *Branchiostoma lanceolatum*. Nature Methods 10, 407–409. 23524392 10.1038/nmeth.2413 PMC3811051

[CIT0028] Shimada TL, Shimada T Hara-Nishimura I 2010 A rapid and non‐destructive screenable marker, FAST, for identifying transformed seeds of *Arabidopsis thaliana*. The Plant Journal 61, 519–528. 19891705 10.1111/j.1365-313X.2009.04060.x

[CIT0029] Sohn KH, Hughes RK, Piquerez SJ, Jones JD, Banfield MJ 2012 Distinct regions of the *Pseudomonas syringae* coiled-coil effector AvrRps4 are required for activation of immunity. Proceedings of the National Academy of Sciences, USA 109, 16371–16376. 10.1073/pnas.1212332109 PMC3479578 22988101

[CIT0030] Sohn KH, Segonzac C, Rallapalli G, Sarris PF, Woo JY, Williams SJ, Newman TE, Paek KH, Kobe B, Jones JD 2014 The nuclear immune receptor RPS4 is required for RRS1SLH1-dependent constitutive defense activation in *Arabidopsis thaliana*. PLoS Genetics 10, e1004655. 25340333 10.1371/journal.pgen.1004655 PMC4207616

[CIT0031] Sohn KH, Zhang Y, Jones JD 2009 The *Pseudomonas syringae* effector protein, AvrRPS4, requires in planta processing and the KRVY domain to function. The Plant Journal 57, 1079–1091. 19054367 10.1111/j.1365-313X.2008.03751.x

[CIT0032] Soleimani VD, Palidwor GA, Ramachandran P, Perkins TJ, Rudnicki MA 2013 Chromatin tandem affinity purification sequencing. Nature Protocols 8, 1525–1534. 23845964 10.1038/nprot.2013.088 PMC4058862

[CIT0033] Thomas WJ, Thireault CA, Kimbrel JA, Chang JH 2009 Recombineering and stable integration of the *Pseudomonas syringae* pv. *syringae* 61 hrp/hrc cluster into the genome of the soil bacterium *Pseudomonas fluorescens* Pf0-1. The Plant Journal 60, 919–928. 19682294 10.1111/j.1365-313X.2009.03998.x

[CIT0034] Tornero P, Chao RA, Luthin WN, Goff SA, Dangl JL 2002 Large-scale structure–function analysis of the Arabidopsis RPM1 disease resistance protein. The Plant Cell 14, 435–450. 11884685 10.1105/tpc.010393 PMC152923

[CIT0034a] Torrens-Spence MP, Bobokalonova A, Carballo V, Glinkerman CM, Pluskal T, Shen A, Weng JK 2019 PBS3 and EPS1 complete salicylic acid biosynthesis from isochorismate in *Arabidopsis*. Molecular Plant 12, P1577–P1586. 10.1016/j.molp.2019.11.005 31760159

[CIT0035] Tsuda K, Mine A, Bethke G, Igarashi D, Botanga CJ, Tsuda Y, Glazebrook J, Sato M, Katagiri F 2013 Dual regulation of gene expression mediated by extended MAPK activation and salicylic acid contributes to robust innate immunity in *Arabidopsis thaliana*. PLoS Genetics 9, e1004015. 24348271 10.1371/journal.pgen.1004015 PMC3861249

[CIT0036] Tsuda K, Sato M, Stoddard T, Glazebrook J, Katagiri F 2009 Network properties of robust immunity in plants. PLoS Genetics 5, e1000772. 20011122 10.1371/journal.pgen.1000772 PMC2782137

[CIT0037] van Wersch R, Li X, Zhang Y 2016 Mighty dwarfs: Arabidopsis autoimmune mutants and their usages in genetic dissection of plant immunity. Frontiers in Plant Science 7, 1717. 27909443 10.3389/fpls.2016.01717 PMC5112265

[CIT0038] Wagner S, Stuttmann J, Rietz S, Guerois R, Brunstein E, Bautor J, Niefind K, Parker JE 2013 Structural basis for signaling by exclusive EDS1 heteromeric complexes with SAG101 or PAD4 in plant innate immunity. Cell Host & Microbe 14, 619–630. 24331460 10.1016/j.chom.2013.11.006

[CIT0039] Wang J, Hu M, Wang J, et al 2019 Reconstitution and structure of a plant NLR resistosome conferring immunity. Science 364, eaav5870. 30948527 10.1126/science.aav5870

[CIT0040] Wickham H 2016 ggplot2—elegant graphics for data analysis. New York: Springer-Verlag New York.

[CIT0041] Williams SJ, Sohn KH, Wan L, et al 2014 Structural basis for assembly and function of a heterodimeric plant immune receptor. Science 344, 299–303. 24744375 10.1126/science.1247357

[CIT0042] Withers J, Dong X 2017 Post-translational regulation of plant immunity. Current Opinion in Plant Biology 38, 124–132. 28538164 10.1016/j.pbi.2017.05.004 PMC5644497

[CIT0043] Wu CH, Abd-El-Haliem A, Bozkurt TO, Belhaj K, Terauchi R, Vossen JH, Kamoun S 2017 NLR network mediates immunity to diverse plant pathogens. Proceedings of the National Academy of Sciences, USA 114, 8113–8118. 10.1073/pnas.1702041114 PMC5544293 28698366

[CIT0044] Wu Z, Li M, Dong OX, Xia S, Liang W, Bao Y, Wasteneys G, Li X 2019 Differential regulation of TNL-mediated immune signaling by redundant helper CNLs. New Phytologist 222, 938–953. 30585636 10.1111/nph.15665

[CIT0045] Yoo H, Greene GH, Yuan M, Xu G, Burton D, Liu L, Marqués J, Dong X 2020 Translational regulation of metabolic dynamics during effector-triggered immunity. Molecular Plant 13, 88–98. 31568832 10.1016/j.molp.2019.09.009 PMC6946852

[CIT0046] Zhang L, Chen S, Ruan J, et al 2015 Cryo-EM structure of the activated NAIP2–NLRC4 inflammasome reveals nucleated polymerization. Science 350, 404–409. 26449474 10.1126/science.aac5789 PMC4640189

[CIT0047] Zhang ZM, Ma KW, Gao L, Hu Z, Schwizer S, Ma W, Song J 2017 Mechanism of host substrate acetylation by a YopJ family effector. Nature Plants 3, 17115. 28737762 10.1038/nplants.2017.115 PMC5546152

[CIT0048] Zuo J, Niu QW, Chua NH 2000 An estrogen receptor-based transactivator XVE mediates highly inducible gene expression in transgenic plants. The Plant Journal 24, 265–273. 11069700 10.1046/j.1365-313x.2000.00868.x

