# Supplementary material for: Estradiol-inducible AvrRps4 expression reveals distinct properties of TIR-NLR-mediated effector-triggered immunity
Source: J Exp Bot. 2020 Feb 12;71(6):2186–97. doi: 10.1093/jxb/erz571 (PMC7242080; doi:10.1093/jxb/erz571)
Supplement: erz571_suppl_Supplementary-Tables-S1-S5_Figures-S1-S3 [file erz571_suppl_supplementary-tables-s1-s5_figures-s1-s3.pdf]

Supplementary Table S1 Information of synthetic promoters used in this study.

Promoter Number	Promoter length (bp)[1]	Gene Symbol (UniProtKB)	AGI (TAIR)	Expression (TPM)[2]
pAt1	1746	*TIP2-1*	AT3G16240	1600
pAt2	711	*RPS16-1*	AT4G34620	850
pAt3	550	*CYSC1*	AT3G61440	600
pAt4	348	*PSBQ1*	AT4G21280	557
pAt5	982	*XTH6*	AT5G65730	1000
pAt6	1659	*UBL5*	AT5G42300	280

1. The promoters are chosen from the first nucleotide next to the start codon ATG on the opposite direction of gene coding direction. The total length of each gene promoter is defined from the start codon of gene of interest up to either the 5’ or 3’ end of the immediate neighbouring gene.
2. TPM, tags per million. Transcripts of each gene under their endogenous promoters are indicated by the RNA sequencing profile data generated in Arabidopsis Ws-2 accession (Sohn *et al.*, 2013).

Supplementary Table S2 Golden Gate stacking construct of R genes and effector.

Position in Level2	Flank[1]	P+5U[2]	CDS or genomic[3]	cTag[4]	Ter[5]	Flank	Simplified Module
1	TGCC	AtOleosin[6]	*AtOleosin*	*RFP*	AtOleosin	GCAA	**FastRed**
2	TTAC	AtActin2	*XVE*		AtuMas[7]	CAGA	**XVE**
3	GCAA	AtSSR16[8]	*AtRRS1-R*	*Hellfire*[9]	AtRRS1-R	ACTA	**RRS1-R-HF**
4	ACTA	AtCysC1[10]	*AtRPS4*	*HA_6*[11]	CaMV35S	TTAC	**RPS4-HA**
5	CAGA	LexA	*PsAvrRps4*[12]	*BlmNeon*[13]	AtuOcs[14]	TGTG	**LexA:AvrRps4-mNeon**
End Linker	TGTG	gaggatgcacatgtgaccga				GGGA	**pELE-5**
Back Bone	TGCC	-				GGGA	**pAGM4723**

1. Flank: the flank sequences indicate the overhang sequence generated by the restriction enzyme BpiI from the level 1 modules to the level 2 destination backbone, before the final ligation reaction.
2. P+5U: promoter and 5' untranslated region (UTR).
3. CDS or genomic: coding sequence or full-length genomic sequence that includes potential introns.
4. cTag: c-terminal in-frame coding sequence for epitope tag.
5. Ter: terminator.
6. AtOleosin: AT4G25140, a protein found in oil bodies, involved in seed lipid accumulation, that is specifically expressed in seed coat.
7: AtuMas: terminator of Mas1 agropine synthesis reductase from *Agrobacterium tumefaciens* (Engler *et al.*, 2014).
8. AtSSR16: SMALL SUBUNIT RIBOSOMAL PROTEIN 16, AT4G34620; was named as pAt2 in our 'moderate promoter' database for intermediate expressing control in transgenic Arabidopsis leaves.
9. HellFire: His_6-TEV-$FLAG_3$, a tandem epitope tag with 6× histidine, TEV protease cleavage site and 3× FLAG tag (Soleimani *et al.*, 2013); here we simplify it as HF.
10. AtCYSC1: CYSTEINE SYNTHASE C1, AT3G61440, was named as pAt3 in our 'moderate promoter' database for intermediate expressing control in transgenic Arabidopsis leaves.
11. HA_6: a tag with 6 tandem HA repeats (Gauss *et al.*, 2005).
12. PsAvrRps4: effector protein AvrRps4 from *Pseudomonas syringae* pv. *pisi*. Here this module can be placed with either wild-type or mutant AvrRps4 coding sequence.
13. BlmNeon: mNeonGreen protein, a bright monomeric green fluorescent protein derived from *Branchiostoma lanceolatum* (Shaner *et al.*, 2013).
14. AtuOcs: terminator of octopine synthase from *Agrobacterium tumefaciens* (Engler *et al.*, 2014).

Supplementary Table S3 Golden Gate cloning modules used in this work.

Modules for Cloning	TSL Synbio Name	Description of Inserts	Backbones	Overhangs for Ligation	
Level 1; Selection Cassettes	pICSL11015	See Table S2 FastRed module	pICH47732	TGCC	GCAA
Level 1; Inducible Cassettes	pICSL11037	See Table S2 XVE module	pICH47742	GCAA	ACTA
Level 0; Promoters + 5' Untranslated Regions (UTRs)	pICSL12028	AtSSR16 promoter, Col-0 allele	-	GGAG	AATG
Level 0; Coding Sequence (CDS) Without A Stop Codon	pICSL80072	*AtRRS1-R* CDS from genomic DNA, Ws-2 allele, BbsI and BpiI sites are removed	-	AATG	TTCG
Level 0; C-terminal Tag	pICSL50001	Hellfire tag, His_6-TEV-$FLAG_3$	-	TTCG	GCTT
Level 0; 3' UTRs and terminators	pICSL60019	AtRRS1-R terminator, Ws-2 allele	-	GCTT	CGCT
Level 0; Promoters + 5' UTRs	pICSL12007	AtCysC1 promoter, Col-0 allele	-	GGAG	AATG
Level 0; CDS Without A Stop Codon	pICSL80073	*AtRPS4* CDS from genomic DNA, Col-0 allele, BbsI and BpiI sites are removed	-	AATG	TTCG
Level 0; C-terminal Tag	pICSL50009	Human influenza hemagglutinin tag, HA_6	-	TTCG	GCTT
Level 0; 3' UTRs and terminators	pICH41414	CaMV 35S terminator	-	GCTT	CGCT
Level 0; Promoters + 5' UTRs	pICSL12005	LexA inducible promoter		GGAG	AATG
Level 0; CDS Without A Stop Codon	pICSL80070	AvrRps4 wild-type coding sequence (CDS) from *Pseudomonas syringae*, BbsI and BpiI sites are removed		AATG	TTCG
Level 0; CDS Without A Stop Codon	pICSL80071	AvrRps4 CDS with KRVY135-138AAAA substitutions, BbsI and BpiI sites are removed		AATG	TTCG
Level 0; CDS Without A Stop Codon	pICSL80074	AvrRps4 CDS with E187A substitution, BbsI and BpiI sites are removed		AATG	TTCG
Level 0; C-terminal Tag	pICSL50015	mNeonGreen fluorescent protein from *Branchiostoma lanceolatum*, BbsI and BpiI sites are removed		TTCG	GCTT
Level 1; Expression Cassettes	pICSL11162	See Table S2 RRS1-R-HF module	pICH47751	ACTA	TTAC
Level 1; Expression Cassettes	pICSL11163	See Table S2 RPS4-HA module	pICH47761	TTAC	CAGA
Level 1; Expression Cassettes	pICSL11164	See Table S2 LexA:AvrRps4-mNeon module	pICH47772	CAGA	TGTG
Level 1; Expression Cassettes	pICSL11165	Similar to pICSL11164, but with KRVY135-138AAAA substitutions	pICH47772	CAGA	TGTG
Level 1; Expression Cassettes	pICSL11166	Similar to pICSL11164, but with E187A substitution	pICH47772	CAGA	TGTG

Supplementary Table S4 Primers used in this study.

Primer Name	Directions	Nucleotide Sequence (5' to 3')
**For real-time quantitative PCR**		
AtEF1α_RT_Fw	forward	CAGGCTGATTGTGCTGTTCTTA
AtEF1α_RT_Rv	reverse	GTTGTATCCGACCTTCTTCAGG
AtICS1_RT_Fw	forward	CAATTGGCAGGGAGACTTACG
AtICS1_RT_Rv	reverse	GAGCTGATCTGATCCCGACTG
AtPR1_RT_Fw	forward	ATACACTCTGGTGGGCCTTACG
AtPR1_RT_Rv	reverse	TACACCTCACTTTGGCACATCC
AvrRps4_RT_Fw	forward	ATGACTCGAATTTCAACC
AvrRps4_RT_Rv	reverse	GGTCCACCCAATAGGGATTTGGGTG
**For cloning**		
AvrRps4_dom_Fw	forward	GAGGGTCTCAAATGACTCGAATTTCAACCAGTTCAG
AvrRps4_dom_Rv	reverse	GAGGGTCTCACGAACCTTGGTTGATTCTGCGGTCCTCG
RPS4_dom_1_Fw	forward	agGAAGACAAAATGGAGACATCATCTATTTCCACTGTGGAgGAC
RPS4_dom_1_Rv	reverse	agGAAGACAAGTCcTCATAGTCGTCGATAAAGAC
RPS4_dom_2_Fw	forward	agGAAGACAAgGACAGAGGTCAACCTCTAGATG
RPS4_dom_2_Rv	reverse	agGAAGACAAGTtTTCACCGCCTTCACAATTTCATTG
RPS4_dom_3_Fw	forward	agGAAGACAAAaACAGCGTTGACCGGAATACCACCGG
RPS4_dom_3_Rv	reverse	agGAAGACAAAtACACTGACAATATTAGGGCTGG
RPS4_dom_4/5_Fw	forward	agGAAGACAAgtattccaagtgagttatgatgaattg
RPS4_dom_4/5_Rv	reverse	agGAAGACAAcctccacttcagacaagtctagg
RPS4_dom_6_Fw	forward	agGAAGACAAGAgGACGAAACGAGCTTAGACCGCGACCAC
RPS4_dom_6_Rv	reverse	agGAAGACAATtTTCAGCGAACTACAGCCGTGTGCATCTAAGC
RPS4_dom_7_Fw	forward	agGAAGACAAAAaACAGTTTCAAAGCCTTTGGCCCGTA
RPS4_dom_7_Rv	reverse	agGAAGACAATaTCTTCATCTTTTACTTTAAAGGTG
RPS4_dom_8_Fw	forward	agGAAGACAAGAtAAGTCTTGGGTCGCATATACTTGTCC
RPS4_dom_8_Rv	reverse	agGAAGACAAGAAtACATGGTCTAGCTCAATCTTATCTTT
RPS4_dom_9_Fw	forward	agGAAGACAAaTTCATTGGATACACCAGTTG
RPS4_dom_9_Rv	reverse	agGAAGACAAcgaaccGAAATTCTTAACCGTGTGCATGA

Supplementary Table S5 Statistical analysis results.

Figure 4C

Tukey multiple comparison of means

1hpi

Condition 1	Condition 2	Diff[1]	Lower[2]	Upper[2]	p adjusted[3]
SETI_eds1 Mock	SETI_eds1 E2	-0.33333330	-3.07588100	2.40921400	0.99814890
SETI_KRVYmut E2	SETI_eds1 E2	-0.33333330	-3.07588100	2.40921400	0.99814890
SETI_KRVYmut Mock	SETI_eds1 E2	-0.66666670	-3.40921400	2.07588100	0.95879800
SETI_WT E2	SETI_eds1 E2	0.00000000	-2.74254700	2.74254700	1.00000000
SETI_WT Mock	SETI_eds1 E2	-1.00000000	-3.74254700	1.74254700	0.81720430
SETI_KRVYmut E2	SETI_eds1 Mock	0.00000000	-2.74254700	2.74254700	1.00000000
SETI_KRVYmut Mock	SETI_eds1 Mock	-0.33333330	-3.07588100	2.40921400	0.99814890
SETI_WT E2	SETI_eds1 Mock	-0.33333330	-3.07588100	2.40921400	0.99814890
SETI_WT Mock	SETI_eds1 Mock	-0.66666670	-3.40921400	2.07588100	0.95879800
SETI_KRVYmut Mock	SETI_KRVYmut E2	-0.33333330	-3.07588100	2.40921400	0.99814890
SETI_WT E2	SETI_KRVYmut E2	-0.33333330	-3.07588100	2.40921400	0.99814890
SETI_WT Mock	SETI_KRVYmut E2	-0.66666670	-3.40921400	2.07588100	0.95879800
SETI_WT E2	SETI_KRVYmut Mock	-0.66666670	-3.40921400	2.07588100	0.95879800
SETI_WT Mock	SETI_KRVYmut Mock	-0.33333330	-3.07588100	2.40921400	0.99814890
SETI_WT Mock	SETI_WT E2	-1.00000000	-3.74254700	1.74254700	0.81720430

5hpi

Condition 1	Condition 2	Diff	Lower	Upper	p adjusted
SETI_eds1 Mock	SETI_eds1 E2	-1.00000000	-8.05166000	6.05166000	0.99617570
SETI_KRVYmut E2	SETI_eds1 E2	-3.00000000	-10.05166000	4.05166000	0.71072430
SETI_KRVYmut Mock	SETI_eds1 E2	-3.33333330	-10.38499300	3.71832700	0.62047410
SETI_WT E2	SETI_eds1 E2	24.33333330	17.28167300	31.38499300	0.00000080
SETI_WT Mock	SETI_eds1 E2	2.33333330	-4.71832700	9.38499300	0.86763760
SETI_KRVYmut E2	SETI_eds1 Mock	-2.00000000	-9.05166000	5.05166000	0.92433520
SETI_KRVYmut Mock	SETI_eds1 Mock	-2.33333330	-9.38499300	4.71832700	0.86763760
SETI_WT E2	SETI_eds1 Mock	25.33333330	18.28167300	32.38499300	0.00000050
SETI_WT Mock	SETI_eds1 Mock	3.33333330	-3.71832700	10.38499300	0.62047410
SETI_KRVYmut Mock	SETI_KRVYmut E2	-0.33333330	-7.38499300	6.71832700	0.99998160
SETI_WT E2	SETI_KRVYmut E2	27.33333330	20.28167300	34.38499300	0.00000020
SETI_WT Mock	SETI_KRVYmut E2	5.33333330	-1.71832700	12.38499300	0.18678740
SETI_WT E2	SETI_KRVYmut Mock	27.66666670	20.61500700	34.71832700	0.00000020
SETI_WT Mock	SETI_KRVYmut Mock	5.66666670	-1.38499300	12.71832700	0.14639040
SETI_WT Mock	SETI_WT E2	-22.00000000	-29.05166000	-14.94834000	0.00000250

20hpi

Condition 1	Condition 2	Diff	Lower	Upper	p adjusted
SETI_eds1 Mock	SETI_eds1 E2	-3.3333333	-12.656202	5.989535	0.8284027
SETI_KRVYmut E2	SETI_eds1 E2	-5.0000000	-14.322868	4.322868	0.4993132
SETI_KRVYmut Mock	SETI_eds1 E2	-5.3333333	-14.656202	3.989535	0.4350794
SETI_WT E2	SETI_eds1 E2	40.3333333	31.010465	49.656202	0.0000001
SETI_WT Mock	SETI_eds1 E2	-0.6666667	-9.989535	8.656202	0.9998581
SETI_KRVYmut E2	SETI_eds1 Mock	-1.6666667	-10.989535	7.656202	0.9889748
SETI_KRVYmut Mock	SETI_eds1 Mock	-2.0000000	-11.322868	7.322868	0.9755368
SETI_WT E2	SETI_eds1 Mock	43.6666667	34.343798	52.989535	0.0000000
SETI_WT Mock	SETI_eds1 Mock	2.6666667	-6.656202	11.989535	0.9218678
SETI_KRVYmut Mock	SETI_KRVYmut E2	-0.3333333	-9.656202	8.989535	0.9999954
SETI_WT E2	SETI_KRVYmut E2	45.3333333	36.010465	54.656202	0.0000000
SETI_WT Mock	SETI_KRVYmut E2	4.3333333	-4.989535	13.656202	0.6357179
SETI_WT E2	SETI_KRVYmut Mock	45.6666667	36.343798	54.989535	0.0000000
SETI_WT Mock	SETI_KRVYmut Mock	4.6666667	-4.656202	13.989535	0.5667788
SETI_WT Mock	SETI_WT E2	-41.0000000	-50.322868	-31.677132	0.0000001

24hpi

Condition 1	Condition 2	Diff	Lower	Upper	p adjusted
SETI_eds1 Mock	SETI_eds1 E2	-3.666667	-13.298177	5.964843	0.7907177
SETI_KRVYmut E2	SETI_eds1 E2	-4.666667	-14.298177	4.964843	0.5976463
SETI_KRVYmut Mock	SETI_eds1 E2	-5.666667	-15.298177	3.964843	0.4072727
SETI_WT E2	SETI_eds1 E2	41.666667	32.035157	51.298177	0.0000001
SETI_WT Mock	SETI_eds1 E2	-1.333333	-10.964843	8.298177	0.9965824
SETI_KRVYmut E2	SETI_eds1 Mock	-1.00000000	-10.63151	8.63151	0.9991284
SETI_KRVYmut Mock	SETI_eds1 Mock	-2.00000000	-11.63151	7.63151	0.9787215
SETI_WT E2	SETI_eds1 Mock	45.333333	35.701823	54.964843	0.0000000
SETI_WT Mock	SETI_eds1 Mock	2.333333	-7.298177	11.964843	0.9593638
SETI_KRVYmut Mock	SETI_KRVYmut E2	-1.00000000	-10.63151	8.63151	0.9991284
SETI_WT E2	SETI_KRVYmut E2	46.333333	36.701823	55.964843	0.0000000
SETI_WT Mock	SETI_KRVYmut E2	3.333333	-6.298177	12.964843	0.8458559
SETI_WT E2	SETI_KRVYmut Mock	47.333333	37.701823	56.964843	0.0000000
SETI_WT Mock	SETI_KRVYmut Mock	4.333333	-5.298177	13.964843	0.664365
SETI_WT Mock	SETI_WT E2	-43.00000000	-52.63151	-33.36849	0.0000000

Figure 5A, B

PR1/EF1A in SETI_WT

Group 1	Group 2	p[4]	p adjusted	p.format[5]	p.signif[6]	method
0h	Untreated	0.0173	0.035	0.0173	*	T-test
2h	Untreated	0.00493	0.028	0.0049	**	T-test
4h	Untreated	0.00258	0.026	0.0026	**	T-test
8h	Untreated	0.00352	0.028	0.0035	**	T-test

PR1/EF1A in SETI_KRVYmut

Group 1	Group 2	p	p adjusted	p.format	p.signif	method
0h	Untreated	0.248	0.25	0.24812	ns	T-test
2h	Untreated	0.062	0.19	0.06204	ns	T-test
4h	Untreated	0.0342	0.17	0.03423	*	T-test
8h	Untreated	0.0191	0.11	0.01912	*	T-test

ICS1/EF1A in SETI_WT

Group 1	Group 2	p	p adjusted	p.format	p.signif	method
0h	Untreated	0.075	0.11	0.07535	ns	T-test
2h	Untreated	0.0096	0.05	0.00969	**	T-test
4h	Untreated	0.00788	0.055	0.00788	**	T-test
8h	Untreated	0.00292	0.023	0.00292	**	T-test

ICS1/EF1A in SETI_KRVYmut

Group 1	Group 2	p	p adjusted	p.format	p.signif	method
0h	Untreated	0.00713	0.057	0.0071	**	T-test
2h	Untreated	0.182	0.36	0.1819	ns	T-test
4h	Untreated	0.0115	0.08	0.0115	*	T-test
8h	Untreated	0.0369	0.17	0.0369	*	T-test

Figure 6

0dpi

Tukey multiple comparison of means

Condition 1	Condition 2	Diff	Lower	Upper	p adjusted
Col-0 Mock	Col-0 E2	-0.009058683	-0.13119346	0.11307609	0.996022
SETI_WT E2	Col-0 E2	-0.031042838	-0.15317762	0.09109194	0.8730022
SETI_WT Mock	Col-0 E2	0.051462467	-0.07067231	0.17359724	0.6085929
SETI_WT E2	Col-0 Mock	-0.021984155	-0.14411893	0.10015062	0.9489591
SETI_WT Mock	Col-0 Mock	0.06052115	-0.06161363	0.18265593	0.4829018
SETI_WT Mock	SETI_WT E2	0.082505305	-0.03962947	0.20464008	0.2391907

T-test

Genotype	Group1	Group2	p	p.adj	p.format	p.signif
Col-0	Col-0 E2	Col-0 Mock	0.852	0.85	0.852	ns
SETI_WT	SETI_WT E2	SETI_WT Mock	0.0676	0.14	0.068	ns

3dpi

Tukey multiple comparison of means

Condition 1	Condition 2	Diff	Lower	Upper	p adjusted
Col-0 Mock	Col-0 E2	-0.08591854	-0.7827627	0.6109256	0.9854528
SETI_WT E2	Col-0 E2	-1.2506235	-1.9474676	-0.5537794	0.0003516
SETI_WT Mock	Col-0 E2	0.19040795	-0.5064362	0.8872521	0.8692549
SETI_WT E2	Col-0 Mock	-1.16470496	-1.8615491	-0.4678608	0.0007702
SETI_WT Mock	Col-0 Mock	0.27632649	-0.4205176	0.9731706	0.6877704
SETI_WT Mock	SETI_WT E2	1.44103146	0.7441873	2.1378756	0.0000637

T-test

Genotype	Group1	Group2	p	p.adj	p.format	p.signif
Col-0	Col-0 E2	Col-0 Mock	0.621	0.62	0.6213	ns
SETI_WT	SETI_WT E2	SETI_WT Mock	0.000905	0.0018	0.0009	***

1. **diff**: difference between means of the two groups
2. **lower**, **upper**: the lower and the upper end point of the confidence interval at 95% (default)
3. **p adjusted**: p-value after adjustment for the multiple comparisons.
4. **p**: p-value
5. **p.format**: formatted p value
6. **p.signif**: significance levels

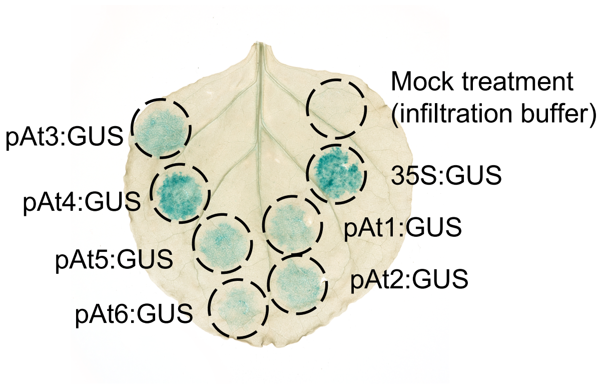

Supplementary Figure S1 GUS-staining activity of synthetic promoters in *N. benthamiana*.

Synthetic promoters At1-At6 were fused to β-glucuronidase (GUS) gene and infiltrated into *N. benthamiana* leaves. GUS expressed under the 35S promoter served as positive control. Mock treatment (infiltration with infiltration buffer) was used as negative control. Leaf samples were collected at 2 days post infiltration (dpi), and GUS staining was performed as in Materials and Methods.

Supplementary Figure S2

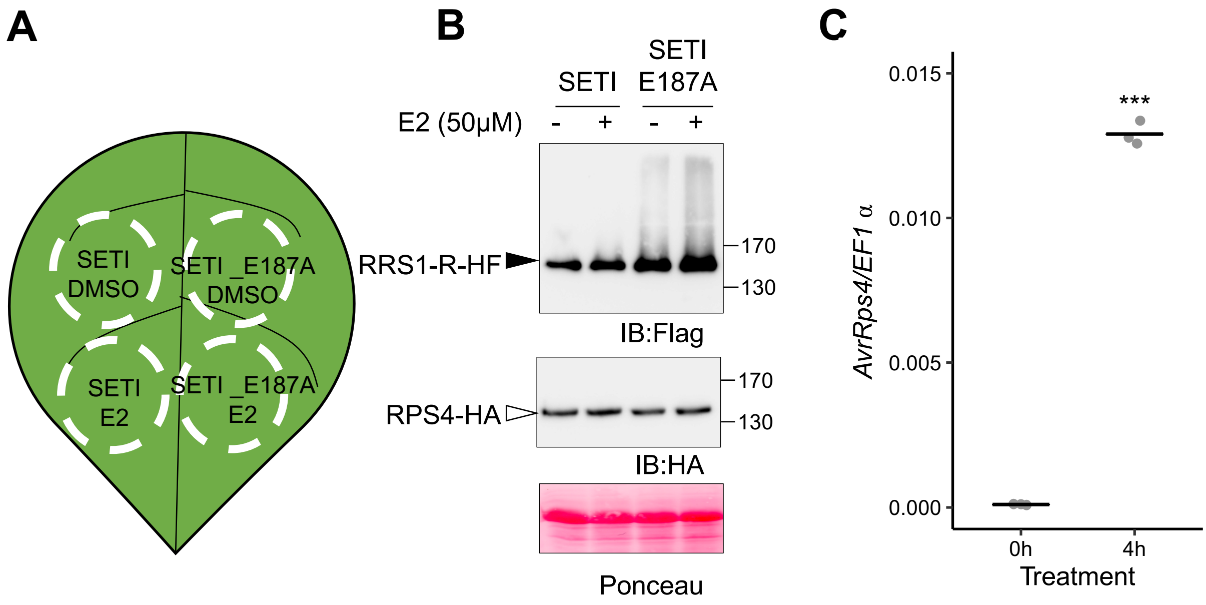

Supplementary Figure S2 Transient expression of Super-ETI (SETI) constructs in *N. benthamiana*.

(A) Schematc diagram of the SETI construct infiltration in *N. benthamiana*. Leaves were infiltrated with Agrobacterium containing SETI or SETI_E187A constructs. At 2dpi, leaves were re-infiltrated with 0.1% DMSO or 50μM E2 according to the diagram. Samples were taken 6h after infiltration with 0.1% DMSO or 50μM E2.

(B) Protein accumulation of RRS1-R-HF and RPS4-HA by transient expression in N. benthamiana. Crude extracts of leaf samples from (A) were immunoblotted with Flag antibody (IB:Flag) to detect RRS1-R-HF (black arrowhead) or HA antibody (IB:HA) to detect RPS4-HA(white arrowhead). Ponceau staining of Rubisco large subunits is the loading control.

(C) *AvrRps4* expression after induction with E2 for 4h in the SETI leaves. 5-week old SETI leaves were infiltrated with 50 μM E2. Samples were collected at 0 and 4hpi for RNA extraction and subsequent qPCR. Expression level is presented as relative to *EF1α* expression. Each data point represents one technical replicate. Black line represents the mean of the technical replicates. This experiment was repeated three times independently with similar results.

Supplementary Figure S3

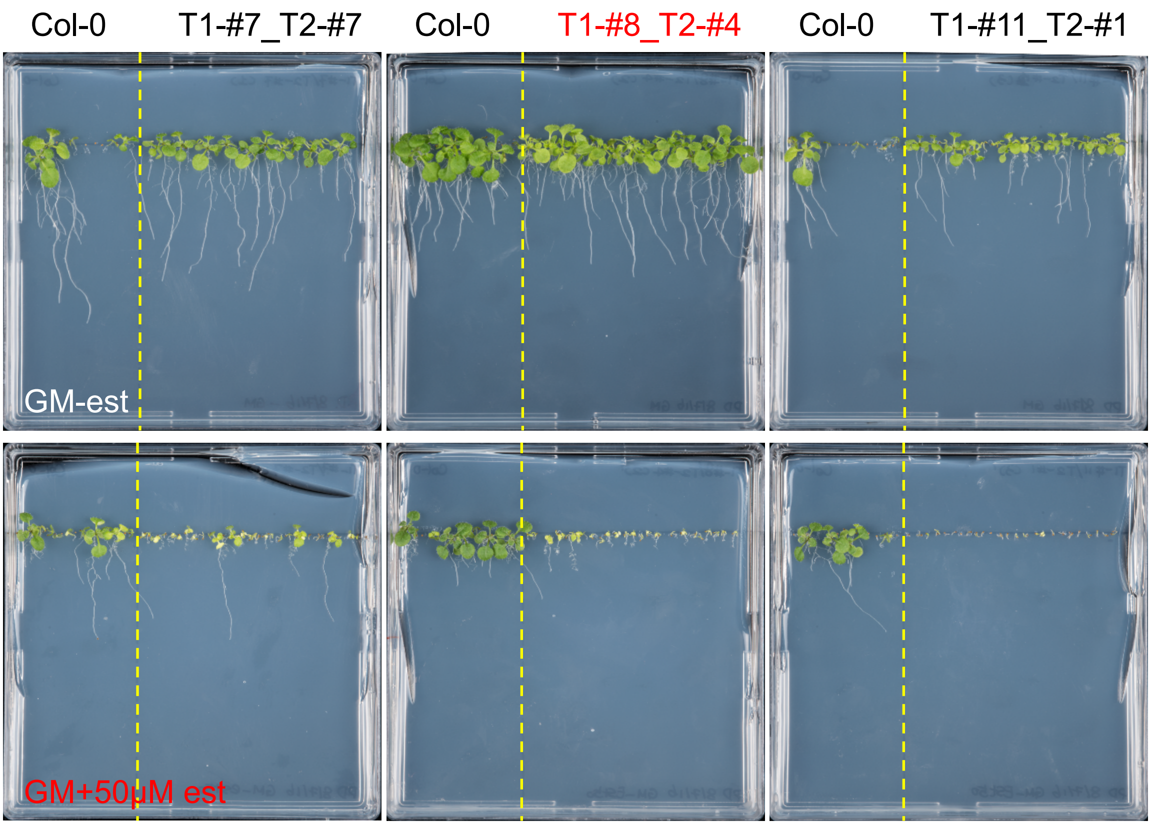

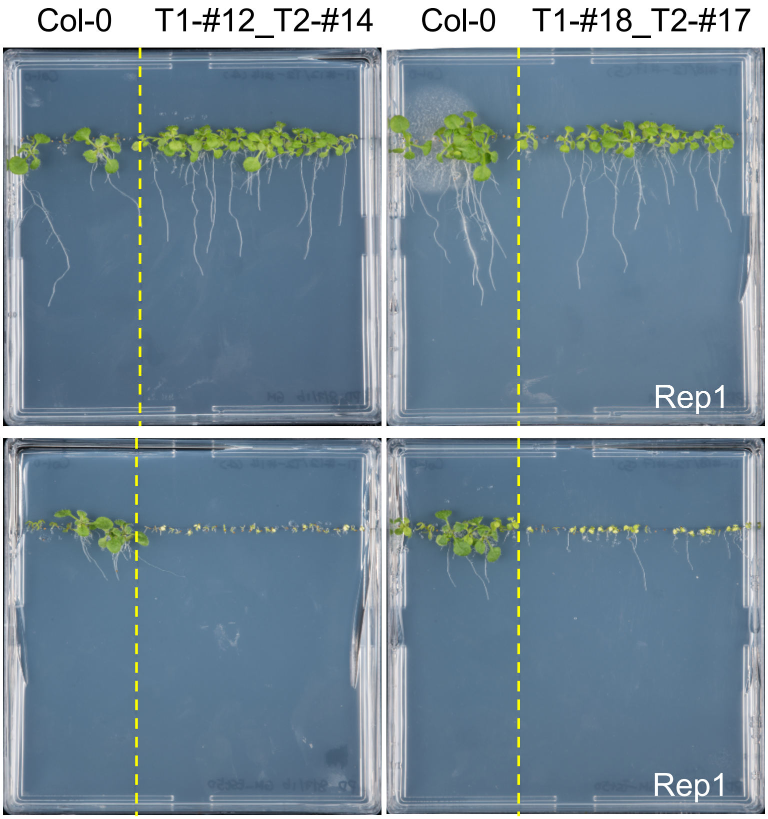

Supplementary Figure S3
SETI T2 lines grown under E2 treatment.

SETI-transformed Arabidopsis transgenic seedlings were sown in GM either containing 50μM E2 or its solvent 0.1% DMSO. Images were taken at 14DAG. Further analysis of SETI lines were performed with the line T1-#8_T2-#4, indicated in red.
